# Books Reviewed

**Published:** 1869-02

**Authors:** 


### Books Reviewed.

The Published Writings of the late Thomas Addison, Physician to Guy's Hospital. New Sydenham Society, 1868.

In this work we have a biography of Dr. Addison, commencing at the time of his first connection with Guy's Hospital.

The published writings are papers upon the following topics:

I.—Observations upon the Anatomy of the Lungs.

II.—Observations on the Diagnosis of Pneumonia.

III.—Observations upon Pneumonia and its Consequences.

IV.—On the Pathology of Phthisis.

V.—On the Difficulties and Fallacies attending Physical Diagnosis in Diseases of the Chest.

VI. Observations on Fatty Degeneration of the Liver.

VII.—On the Disorders of Females connected with Uterine Irritation.

VIII.—Case of Ovarian Dropsy removed by the accidental rupture of the Cyst.

IX.—On a certain Affection of the Skin, Vitiligoidea.

X.—On the Keloid or Alibert and on true Keloid.

XI.—On Disorders of the Brain connected with Diseased Kidneys.

XII.—On the Influence of Electricity in Convulsive and Spasmodic Diseases.

XIII.—On the Constitutional and Local Effects of Disease of the Supra-renal Capsules.

These papers are all of value, containing results of original investigation, and much thought. Some of them are illustrated in an elegant manner, and thus the text is more easily and more fully comprehended.

The illustrations of diseased conditions of the lungs, which are rare, are truly very superior. The writings of Dr. Addison show how faithfully he studied disease, and how watchfully he guarded his opinions. He was in one of the best schools for observation and did not waste or neglect his opportunity.

---

Hebra on Diseases of the Skin. Vol. II. New Sydenham Society, 1868.

The second volume of this work treats upon Psoriasis; Lichen; Pityriasis Rubra; Eczema; Scabies; Prurigo; Acne Disseminata; Sycosis; Acne Rosacea; Pustula Eruptions; Pemphigus; Rupia, and Hæmorrhagiæ Cutaneæ.

These diseases are considered under the following heads: history, definition, varieties, course, diagnosis, etiology, prognosis, anatomy, treatment.

The work is written in clear, terse and elegant manner, and embodies all that is known upon the various diseases in question. Perhaps it is not saying too much when we express the opinion that no where can be found a more satisfactory account of these various skin diseases than in this work.

---

Lectures on the Study of Fever. By Alfred Hudson, M. D. Philadelphia: Henry C. Lea, 1869.

We have examined the teaching of these lectures with some care, and find that the philosophy of fever, the varieties, the distinctions, the symptoms and the pathological conditions, are all presented to the reader in unexceptionable manner, so far as present knowledge can permit. The suggestions for treatment are very numerous, and appear to us to favor an activity and interference wholly unsustained by what is known of these diseases—of their natural progress and tendencies, and of the effects of remedial agents.

Pathological Anatomy of the Female Sexual Organs. By Julius M. Klob, M. D. Wm. Wood & Co., 1868.

This is a work upon the various anomalies of the uterus, and comprises a consideration of all the diseased conditions usually treated in works upon uterine diseases. We have not examined it carefully enough to discover anything especially new, but observe that it is in a style somewhat peculiar to the author, yet embodying a scientific and very careful investigation of the various conditions of disease and displacements common, and uncommon, to this organ. The pathology of the diseases of the uterus is presented more fully and to more recent date than almost anywhere else in similar works, and on this account mainly do we earnestly recommend it to the careful study of all interested in the study of diseases of the uterus.

---

Practical Observations on the Ætiology, Pathology, Diagnosis and Treatment of Anal Fissure. By William Bodenhamer, A. M., M. D.

The author informs us that the disease termed *fissure of the anus*, is not well understood; the diagnosis not established upon a solid basis; that there is no agreement as to precise meaning of the term, and great diversity as to the best method of cure. The object of the work appears to be, to remove some of the obscurities, the difficulties and the confusion which surround it. Besides, the author is led to call attention to the subject of this disease because there is no systematic treatise upon it and its exceedingly practical importance from its frequency and the great suffering to which it gives rise. So far as the author's treatment is concerned, he says he has nothing new to offer in this work. We believe that the subject has received full justice at the hands of the author, and that the work will be a standard authority upon the subject.

---

Notice of a set of Instruments, arranged by Surgeon P. S. Wales, U. S. N., (author of the recent work on "Mechanical Therapeutics,") and manufactured by J. H. Gemrig, 109 S. 8th street, Philadelphia. By H. P. Babcock, M. D., late U. S. N., now of Pacific Mail Co.'s service.

These are the most complete cases we have ever seen, both as to selection of instruments, compactness of arrangement, beauty of finish and price. The fact of their coming from Gemrig is sufficient evidence that they are in material and workmanship, as perfect as art can make them. To practitioners, living away from large cities, and where, in emergencies, instruments cannot be at once obtained, these cases are particnlarly valuable as containing everything one is likely to require, and with an obstetric set would be a complete outfit. We give their contents.

"WALES' CAPITAL OPERATING CASE."

*In top.*—One large amputating saw—two blades; one chain saw; one Hey's saw; one Liston's large bone forceps, with spring; one sequestrum forceps, with spring; one artery continued-pressure forceps; one lithotomy forceps; two conical trephines; one elevator; one brush; four of Brainerd's drills, with handle; one gouge; one chisel; one needle-book, ten assorted needles; silk; silver wire; wax, etc.

*In bottom.*—Two long, one short amputating knives; one catlin; one saw, movable back; one bone knife; two scalpels; one bistoury; one tenaculum; one tourniquet; one small bone forceps, with spring; one gnawing forceps, with spring; two steel sounds; two lithotomy staffs, direct groove; one Heurteloup's rack and pinion lithotrite. The case of mahogany, brass-bound, lock and key, size, 17 inches by 9¼

inches, by $3\frac{1}{4}$ inches deep, enclosed in sole leather case, lined with canton flannel. Price $112.00.

"WALES' MINOR OPERATING CASE."

*In top.*—Six steel bougies, silvered, double curve, Nos. 1 to 12; four silver catheters, No. 5, 7, 9, 11; six gum-elastic catheters, No. 1, 3, 5, 7, 9, 10; one double trachea tube; one paper steel pins; one needle-book, eight assorted needles; silk; silver wire; wax, etc.

*In tray.*—One Bond's æsophageal forceps; one Gemrig's bullet forceps; one polypus forceps; one Museux's forceps; one dilating forceps; one needle and artery forceps; one plain forceps; one Bellocq's canula; one double canula; three pair scissors, plain, curved on flat, angular; one Nealton's probe; one grooved director; one Mott's artery needle, three points and blunt hook; one tenaculum; one hernia knife; two curved bistouries, sharp and probe; one finger knife; four scalpels; one tenotomy knife; one scarifying knife.

*In bottom.*—One bone gnawing forceps; one small, straight bone forceps; one curved and one straight trocar and canula; two retractors; one Wales' combination opthalmoscope, laryngoscope, otoscope and endoscope, with head band; comprising one large and one small mirror fitted with three Toynbee's ear specula; one rectal, two urethral specula; three extra lenses; caustic holder; scarifying knife; two laryngeal mirrors, with handle; two sponge holders. The case is of mahogany, brass-bound, lock and key, size 16 inches by 9 inches, by $4\frac{1}{2}$ inches deep, enclosed in sole leather case, lined with canton flannel. Price $150.00.

"WALES' EYE AND EAR CASE."

*In tray.*—Three pair scissors, straight, curved on flat, angular strabismus; one iris scissors; two iris forceps, curved and straight; one cilia forceps; two cataract needles; one curette; one Tyrrel's hook; one cystitome; one Critchett's spoon; one iris knife; one strabismus hook; one sharp hook; one scoop and hook for foreign bodies; one broad needle; one Beer's cataract knife.

*In bottom.*—One Anel's syringe, with two terminal tubes; one eustachian catheter; one Toynbee's ear forceps; one Liebrich's opthalmoscope, with two convex, five concave lenses; one Laurence's speculum; two silver styles; one set Bowman's probes, assorted and numbered; one needle-book, with six needles, assorted; silk; silver wire; wax, etc. Case mahogany, brass bound, lock and key, size $9\frac{3}{4}$ inches by 6 inches, by 3 inches deep, enclosed in sole leather case, lined with canton flannel. Price $70.00.

